# Supplementary material for: Several different sequences are implicated in bloodstream-form-specific gene expression in Trypanosoma brucei
Source: PLoS Negl Trop Dis. 2022 Mar 21;16(3):e0010030. doi: 10.1371/journal.pntd.0010030 (PMC8982893; doi:10.1371/journal.pntd.0010030)
Supplement: S1 Fig — The sequences were aligned using the Segman programme in the DNAStar package. (PDF) [file pntd.0010030.s003.pdf]

```
                      10        20        30        40        50        60
                      ACTTCCAGAAAAAATATATTTCTGCAAAATACTTTTGGAAGTTTGTCTTGTCTTTATAGATGAAGGAT
PGKC_FL(1>780)        ACTTCCAGAAAAAATATATTTCTGCAAAATACTTTTGGAAGTTTGTCTTGTCTTTATAGATGAAGGAT
PGK1(1>781)           ACTTCCAGAAAAAATATATTTCTGCAAAATACTTTTGGAAGTTTGTCTTGTCTTTATAGATGAAGGAT
PGK6_1(1>653)         ACTTCCAGAAAANATATATTTCTGCAAAATACTTTTGGAAGTTTGTCTTGTCTTTATAGATGAAGGAT
PGK7_2(1>500)         ACTTCCAGAAAAAATATATTTCTGCACAATACTTTTGGAAGTTTGTCTTGTCTTTATAGATGAAGGAT
PGK8_1(1>392)         ACTTCCAGAANAAATATATTTCTGCAAAATACTTTTGGAAGTTTGTCTTGTCTTTATAGATGAAGGAT
PGK9(1>294)           ACTTCCAGAAAAAATATATTTCTGCAAAATACTTTTGGAAGTTTGTCTTGTCTTTATAGATGAAGGAT
PGK10(1>102)          ACTTCCAGAAAAAATATATTTCTGCAAAATACTTTTGGAAGTTTGTCTTGTCTTTATAGATGAAGGAT

                      70        80        90        100       110       120       130
                      TTGTTTCTTTTTTGTTTGTGATGTTTTCAAGGTTAATTAGTTTTGGGGGTTCGTTATCTTAATTATTT
PGKC_FL(1>780)        TTGTTTCTTTTTTGTTTGTGATGTTTTCAAGGTTAATTAGTTTTGGGGGTTCGTTATCTTAATTATTT
PGK1(1>781)           TTGTTTCTTTTTTGTTTGTGATGTTTTCAAGGTTAATTAGTTTTGGGGGTTCGTTATCTTAATTATTT
PGK2_1(1>679)                                         AATTAGTTTTGGGGGTTCGTTATCTTAATTATTT
PGK6_1(1>653)         TTGTTTCTTTTTTGTTTGTGATGTTTTCAAGGTTAATTAGTTTTGGGGGTTCGTTATCTTAATTATTT
PGK7_2(1>500)         TTGTTTCTTTTTTGTTTGTGATGTTTTCAAGGTTAATTAGTTTTGGGGGTTCGTTATCTTAATTATTT
PGK8_1(1>392)         TTGTTTCTTTTTTGT--G--AKGTTTTCAAGGTTARTTARTTTTGGGGGTTCGTTATCTTAATTATTT
PGK9(1>294)           TTGTTTCTTTTTTGTTTGTGATGTTTTCAAGGTTAATTAGTTTTGGGGGTTCGTTATCTTAATTATTT
PGK10(1>102)          TTGTTTCTTTTTTGTTTGTGATGTTTTCAAGGTT
PGK11(1>190)                                          AATTAGTTTTGGGGGTTCGTTATCTTAATTATTT

                      140       150       160       170       180       190       200
                      CGGTGGGTGTGAGTAAATAAAGCAGAGAGGTAAATTTTTTGGTGACACAAAAATTGGGAAGCTTCGTG
PGKC_FL(1>780)        CGGTGGGTGTGAGTAAATAAAGCAGAGAGGTAAATTTTTTGGTGACACAAAAATTGGGAAGCTTCGTG
PGK1(1>781)           CGGTGGGTGTGAGTAAATAAAGCAGAGAGGTAAATTTTTTGGTGACACAAAAATTGGGAAGCTTCGTG
PGK2_1(1>679)         CGGTGGGTGTGAGTAAATAAAGCAGAGAGGTAAATTTTTTGGTGACACAAAAATTGGGAAGCTTCGTG
PGK6_1(1>653)         CGGTGGGTGTGAGTAAATAAAGCAGAGAGGTAAATTTTTTGGTGACACAAAAATTGGGAAGCTTCGTG
PGK7_2(1>500)         CGGTGGGTGTGAGTAAATAAAGCAGAGAGGTAAATTTTTTGGTGACACAAAAATTGGGAAGCTTCGTG
PGK8_1(1>392)         TGGTGGGTGGGAGTAAATAAAGCAR--AGGTAAATTTTTTGGTGACACAAAAATTGGGAAGCTTCSTG
PGK9(1>294)           CGGTGGGTGTGAGTAAATAAAGCAGAGAGGTAAATTTTTTGGTGACACAAAAATTGGGAAGCTTCGTG
PGK11(1>190)          TGGTGGGTGGGAGTAAATAAAGC--AGAGGTAAATTTTTTGGTGACACAAAAATTGGGAAGCTTCGTG

                      210       220       230       240       250       260       270
                      TTCTTACTTGTTCAACTGAAAAATGCCTTTTCAGGAATTCATATTTGGGAGTTATTGTGGTGTAGAAG
PGKC_FL(1>780)        TTCTTACTTGTTCAACTGAAAAATGCCTTTTCAGGAATTCATATTTGGGAGTTATTGTGGTGTAGAAG
PGK1(1>781)           TTCTTACTTGTTCAACTGAAAAATGCCTTTTCAGGAATTCATATTTGGGAGTTATTGTGGTGTAGAAG
PGK2_1(1>679)         TTCTTACTTGTTCAACTGAAAAATGCCTTTTCAGGAATTCATATTTGGGAGTTATTGTGGTGTAGAAG
PGK6_1(1>653)         TTCTTACTTGTTCAACTGAAAAATGCCTTTTCAGGAATTCATATTTGGGAGTTATTGTGGTGTAGAAG
PGK7_2(1>500)         TTCTTACTTGTTCAACTGAAAAATGCCTTTTCAGGAATTCATATTTGGGAGTTATTGTGGTGTAGAAG
PGK8_1(1>392)         TTCTTACTTGTTCAACTGAAAAATGCCTTTTCAGGAATTCATATTTGGGAGTTATTGTGGKGTAGAAG
PGK9(1>294)           TTCTTACTTGTTCAACTGAAAAATGCCTTTTCAGGAATTCATATTTGGGAGTTATTGTGGTGTAGAAG
PGK11(1>190)          TTCTTACTTGTTCAACTGAAAAATGCCTTTTCAGGAATTCATATTTGGGAGTTATTGTGGTGTAGAAG

                      280       290       300       310       320       330       340
                      GACTGAGGAACAGAAGAAAGCAGAGGTTATTTGCCCCTTCATGAGGAAATGTCGATGTAATTAAGTAT
PGKC_FL(1>780)        GACTGAGGAACAGAAGAAAGCAGAGGTTATTTGCCCCTTCATGAGGAAATGTCGATGTAATTAAGTAT
PGK1(1>781)           GACTGAGGAACAGAAGAAAGCAGAGGTTATTTGCCCCTTCATGAGGAAATGTCGATGTAATTAAGTAT
PGK2_1(1>679)         GACTGAGGAACAGAAGAAAGCAGAGGTTATTTGCCCCTTCATGAGGAAATGTCGATGTAATTAAGTAT
PGK3(1>485)                                 GAGGTTATTTGCCCCTTCATGAGGAAATGTCGATGTAATTAAGTAT
PGK6_1(1>653)         GACTGAGGAACAGAAGAAAGCAGAGGTTATTTGCCCCTTCATGAGGAAATGTCGATGTAATTAAGTAT
PGK7_2(1>500)         GACTGAGGAACAGAAGAAAGCAGAGGTTATTTGCCCCTTCATGAGGAAATGTCGATGTAATTAAGTAT
PGK8_1(1>392)         GACTGAGGAACAGAAGAAAGMARAGGTWATTTGCCCCTTCMTGAGGAAATGTCGATGTAATTAAGTAT
PGK9(1>294)           GACTGAGGAACAGAAGAAAGCA
PGK11(1>190)          GACTGAGGAACAGAAGAAAGCA
PGK12(1>104)                                GAGGTTATTTGCCCCTTCATGAGGAAATGTCGATGTAATTAAGTAT
```

```
                          350       360       370       380       390       400
                     GAGGGAGGACATGTTGATRCTGGGAAATGRACTCTAAAAATGAGAAATAAAGGGAAAGAGAAAGGAAG
PGKC_FL(1>780)  →    GAGGGAGGACATGTTGATGCTGGGAAATGAACTCTAAAAATGAGAAATAAAGGGAAAGAGAAAGGAAG
PGK1(1>781) ....→    GAGGGAGGACATGTTGATGCTGGGAAATGAACTCTAAAAATGAGAAATAAAGGGAAAGAGAAAGGAAG
PGK2_1(1>679)   →    GAGGGAGGACATGTTGATACTGGGAAATGGACTCTAAAAATGAGAAATAAAGGGAAAGAGAAAGGAAG
PGK3(1>485) ....→    GAGGGAGGACATGTTGATACTGGGAAATGGACTCTAAAAATGAGAAATAAAGGGAAAGAGAAAGGAAG
PGK4_1(1>382)   → ..........................................................AGAAAGGAAG
PGK6_1(1>653)   →    GAGGGAGGACATGTTGATGCTGGGAAATGAACTCTAAAAATGAGAAATAAAGGGAAAGAGAAAGGAAG
PGK7_2(1>500)   →    GAGGGAGGACATGTTGATACTGGGAAATGGACTCTAAAAATGAGAAATAAAGGGAAAGAGAAAGGAAG
PGK8_1(1>392)   →    GAGGGAGGACATGTTGATACTGGGAAATGGACTCTAAAAATGAGAAATAAAGGGAAAG
PGK12(1>104) ...→    GAGGGAGGACATGTTGATACTGGGAAATGGACTCTAAAAATGAGAAATAAAGGGAAAG
PGK16(1>255) ...→ ..........................................................AGAAAGGAAG

                     410       420       430       440       450       460       470
                     AGTGATATATATAT-TTTTTGGAAAAAAAAACACTTTTCTTTTGCTTGCGCTGCTGAGTGGGAGATCA
PGKC_FL(1>780)  →    AGTGATATATATAT-TTTTTGGAAAAAAAAACACTTTTCTTTTGCTTGCGCTGCTGAGTGGGAGATCA
PGK1(1>781) ....→    AGTGATATATATAT-TTTTTGGAAAAAAAAACACTTTTCTTTTGCTTGCGCTGCTGAGTGGGAGATCA
PGK2_1(1>679)   →    AGTGATATATATAT-TTTTTGGAAAAAAAAACACTTTTCTTTTGCTTGCGCTGCTGAGTGGGAGATCA
PGK3(1>485) ....→    AGTGATATATATTTATTTTTGGAAAAAAA--CACCTTTCGTTTGCTTGCGCTGCTGAGTGGGAGATCA
PGK4_1(1>382)   →    AGTGATATATATAT-TTTTTGGAAAAAAAAACACTTTTCTTTTGCTTGCGCTGCTGAGTGGGAGATCA
PGK6_1(1>653)   →    AGTGATATATATAT-TTTTTGGAAAAAAAAACACTTTTCTTTTGCTTGCGCTGCTGAGTGGGAGATCA
PGK7_2(1>500)   →    AGTGATATATATAT-TTTTTGGAAAAAAAAACACTTTTCTTTTGCTTGCGCTGCTGAGTGGGAGATCA
PGK16(1>255) ...→    AGTGATATATATAT-TTTTTGGAAAAAAAAACACTTTTCTTTTGCTTGCGCTGCTGAGTGGGAGATCA

                       480       490       500       510       520       530       540
                     TTCTCCGTGTTATATGTCCTTTTTCTAGTGGTTGAGATTGTGTTGTTGTTTTTTCAATTTCTTCTGTG
PGKC_FL(1>780)  →    TTCTCCGTGTTATATGTCCTTTTTCTAGTGGTTGAGATTGTGTTGTTGTTTTTTCAATTTCTTCTGTG
PGK1(1>781) ....→    TTCTCCGTGTTATATGTCCTTTTTCTAGTGGTTGAGATTGTGTTGTTGTTTTTTCAATTTCTTCTGTG
PGK2_1(1>679)   →    TTCTCCGTGTTATATGTCCTTTTTCTAGTGGTTGAGATTGTGTTGTTGTTTTTTCAATTTCTTCTGTG
PGK3(1>485) ....→    TTCTCCGTGTTATATGTCCTTTTTCTAGTGGTTGAGATTGTGTTGTTGTTTTTTCAATTTCTTCTGTG
PGK4_1(1>382)   →    TTCTCCGTGTTATATGTCCTTTTTCTAGTGGTTGAGATTGTGTTGTTGTTTTTTCAATTTCTTCTGTG
PGK5_1(1>280)   → ..........................TAGTGGTTGAGATTGTGTTGTTGTTTTTTCAATTTCTTCTGTG
PGK6_1(1>653)   →    TTCTCCGTGTTATATGTCCTTTTTCTAGTGGTTGAGATTGTGTTGTTGTTTTTTCAATTTCTTCTGTG
PGK7_2(1>500)   →    TTCTCCGTGTTATATGTCCTTTTTC
PGK14(1>153) ...→ ..........................TAGTGGTTGAGATTGTGTTGTTGTTTTTTCAATTTCTTCTGTG
PGK16(1>255) ...→    TTCTCCGTGTTATATGTCCTTTTTCTAGTGGTTGAGATTGTGTTGTTGTTTTTTCAATTTCTTCTGTG

                         550       560       570       580       590       600       610
                     GATAATCTTCCTCGTGAAGAAGACGCAGAAAGCGGGCCACACGGAGTGAATTCATACCTTACTTAAAA
PGKC_FL(1>780)  →    GATAATCTTCCTCGTGAAGAAGACGCAGAAAGCGGGCCACACGGAGTGAATTCATACCTTACTTAAAA
PGK1(1>781) ....→    GATAATCTTCCTCGTGAAGAAGACGCAGAAAGCGGGCCACACGGAGTGAATTCATACCTTACTTAAAA
PGK2_1(1>679)   →    GATAATCTTCCTCGTGAAGAAGACGCAGAAAGCGGGCCACACGGAGTGAATTCATACCTTACTTAAAA
PGK3(1>485) ....→    GATAATCTTCCTCGTGAAGAAGACGCAGAAAGCGGGCCACACGGAGTGAATTCATACCTTACTTAAAA
PGK4_1(1>382)   →    GATAATCTTCCTCGTGAAGAAGACGCAGAAAGCGGGCCACACGGAGTGAATTCATACCTTACTTAAAA
PGK5_1(1>280)   →    GATAATCTTCCTCGTGAAGAAGACGCAGAAAGCGGGCCACACGGAGTGAATTCATACCTTACTTAAAA
PGK6_1(1>653)   →    GATAATCTTCCTCGTGAAGAAGACGCAGAAAGCGGGCCACACGGAGTGAATTCATACCTTACTTAAAA
PGK14(1>153) ...→    GATAATCTTCCTCGTGAAGAAGACGCAGAAAGCGGGCCACACGGAGTGAATTCATACCTTACTTAAAA
PGK16(1>255) ...→    GATAATCTTCCTCGTGAAGAAGACGCAGAGAGCGGGCCACACGGAGTGAATTCATACCTTACTTAAAA

                           620       630       640       650       660       670       680
                     TAATATAAAACGCATTAAAATATGTAATTATATTTATATATTTTTTTCCCTTTCTTTCTTTTAAAAAA
PGKC_FL(1>780)  →    TAATATAAAACGCATTAAAATATGTAATTATATTTATATATTTTTTTCCCTTTCTTTCTTTTAAAAAA
PGK1(1>781) ....→    TAATATAAAACGCATTAAAATATGTAATTATATTTATATATTTTTTTCCCTTTCTTTCTTTTAAAAAA
PGK2_1(1>679)   →    TAATATAAAACGCATTAAAATATGTAATTATATTTATATATTTTTTTCCCTTTCTTTCTTTTAAAAAA
PGK3(1>485) ....→    TAATATAAAACGCATTAAAATATGTAATTATATTTATATATTTTTTTCCCTTTCTTTCTTTTAAAAAA
PGK4_1(1>382)   →    TAATATAAAACGCATTAAAATATGTAATTATATTTATATATTTTTTTCCCTTTCTTTCTTTTAAAAAA
PGK5_1(1>280)   →    TAATATAAAACGCATTAAAATATGTAATTATATTTATATATTTTTTTCCCTTTCTTTCTTTTAAAAAA
PGK6_1(1>653)   →    TAATATAAAACGCATTAAAATATGTAATTATATTTATATATT
```

```
                          620       630       640       650       660       670       680
                     TAATATAAAACGCATTAAAATATGTAATTATATTTATATATTTTTTTCCCTTTCTTTCTTTTAAAAAA
PGK14(1>153)     →   TAATATAAAACGCATTAAAATATGTAATTATATTTATATATT
PGK15-1(1>132)   →                                      ATCTAGATTTTTCCCTTTCTTTCTTTTAAAAAA
PGK16(1>255)     →   TAATATAAAACGCATTAAAATATGTAATTATATTTATATATT

                            690       700       710       720       730       740
                     AAATTCTCTTTTGTGCTTCTTGCTTCTCTCGTTTTCTAAACTGGGCAATTAATATGCTCGAAAGTAAA
PGKC_FL(1>780)   →   AAATTCTCTTTTGTGCTTCTTGCTTCTCTCGTTTTCTAAACTGGGCAATTAATATGCTCGAAAGTAAA
PGK1(1>781)      →   AAATTCTCTTTTGTGCTTCTTGCTTCTCTCGTTTTCTAAACTGGGCAATTAATATGCTCGAAAGTAAA
PGK2_1(1>679)    →   AAATTCTCTTTTGTGCTTCTTGCTTCTCTCGTTTTCTAAACTGGGCAATTAATATGCTCGAAAGTAAA
PGK3(1>485)      →   AAATTCTCTTTTGTGCTTCTTGCTTCTCTCGTTTTCTAAACTGGGCAATTAATATGCTCGAAAGTAAA
PGK4_1(1>382)    →   AAATTCTCTTTTGTGCTTCTTGCTTCTCTCGTTTTCTAAACTGGGCAATTAATATGCTCGAAAGTAAA
PGK5_1(1>280)    →   AAATTCTCTTTTGTGCTTCTTGCTTCTCTCGTTTTCTAAACTGGGCAATTAATATGCTCGAAAGTAAA
PGK15-1(1>132)   →   TC--TCTCTTTTGTGCTTCTTGCTTCTCTCATTTTCTAAACTGGGCAATTAATATGCTCGAAAGTAAA

                     750       760       770       780
                     TATTGAGGTTATTGAAGAGGGTTGGGG-TGTGAAG
PGKC_FL(1>780)   →   TATTGAGGTTATTGAAGAGGGTTGGGG-TGTGAA
PGK1(1>781)      →   TATTGAGGTTATTGAAGAGGGTTGGGG-TGTGAAG
PGK2_1(1>679)    →   TATTGAGGTTATTGAAGAGGGTTGGGGGTGTGAA
PGK3(1>485)      →   TATTGAGGTTATTGAAGAGGGTTGGGG-TGTGAA
PGK4_1(1>382)    →   TATTGAGGTTATTGAAGAGGGTTGGGG-TGTGAA
PGK5_1(1>280)    →   TATTGAGGTTATTGAAGAGGGTTGGGG-TGTGAA
PGK15-1(1>132)   →   TATTGAGGTTATTGAAGAGGGTTGGGG-TGTGAA
```
