# Supplementary material for: Several different sequences are implicated in bloodstream-form-specific gene expression in Trypanosoma brucei
Source: PLoS Negl Trop Dis. 2022 Mar 21;16(3):e0010030. doi: 10.1371/journal.pntd.0010030 (PMC8982893; doi:10.1371/journal.pntd.0010030)
Supplement: S2 Text — (DOCX) [file pntd.0010030.s005.docx]

PGKC 3'-UTR

**ACUUCCAGAAAAAAUAUAUUUCUGCAAAAUACUUUUGGAAGUUUGUCUUGUCUUUAUAGAUGAAGGAUUUGUUUC**

**............1**

**UUUUUUGUUUGUGA|UGUUUUCAAGGUU*AAUUAGUUUUGGGGGUUCGUUAUCUUAAUUAUUUCGGUGGGUGUGA**

**....................12................2**

**GUAAAUAAAGCAGAGAGGUAAAUUUUUUGGUGACACAAAAAUUGGGAAGCUUCGUGUUCUUACUUGUUCAACUGA**

**AAAAUGCCUUUUCAGGAAUUCAUAUUUGGGAGUUAUUGUGGUGUAGAAGGACUGAGGAACAGAAGAAAGCA*GA**

**............................................................11**

**GGUUAUUUGCCCCUUCAUGAGGAAAUGUCGAUGUAAUUAAGUAUGAGGGAGGACAUGUUGAUGCUGGGAAAUGAA**

**....3**

**CUCUAAAAAUGAGAAAUAAAGGGAAAG*AGAAAGGAAGAGUGAUAUAUAUAUUUUUUGGAAAAAAAAACACUUU**

**.............10.....................4**

**UCUUUUGCUUGCGCUGCUGAGUGGGAGAUCAUUCUCCGUGUUAUAUGUCCUUUUUC*UAGUGGUUGAGAUUGUGU**

**............................................9......................5**

**UGUUGUUUUUUCAAUUUCUUCUGUGGAUAAUCUUCCUCGUGAAGAAGACGCAGAAAGCGGGCCACACGGAGUGAA**

**..............................14**

**UUCAUACCUUACUUAAAAUAAUAUAAAACGCAUUAAAAUAUGUAAUUAUAUUUAUAUAUU|*UUUUUCCCUUUCU**

**13.........................................8...........................6**

**UUCUUUUAAAAAAAAAUUCUCUUUUGUGCUUCUUGCUUCUCUCGUUUUCUAAACUGGGCAAUUAAUAUGCUCGAA**

**AGUAAAUAUUGAGGUUAUUGAAGAGGGUUGGGGUGUGAA**

**..............................7**

KEY

forward primers

reverse primers

| Centroid stem-loops and MFE region boundaries

*cloning boundaries
