# Supplementary material for: Several different sequences are implicated in bloodstream-form-specific gene expression in Trypanosoma brucei
Source: PLoS Negl Trop Dis. 2022 Mar 21;16(3):e0010030. doi: 10.1371/journal.pntd.0010030 (PMC8982893; doi:10.1371/journal.pntd.0010030)
Supplement: Attachment — Submitted filename: _Reply to reviewers_21_2.docx [file pntd.0010030.s008.docx]

We thank the reviewers for their constructive suggestions and for finding our errors. We have now provided all requested information and data, and made corrections as suggested. In the text below our answers are in red.

I have evaluated the manuscript and the comments of the three expert reviewers. This work has clear merit and will contribute to our understanding of the mechanisms of gene expression regulation in trypanosomes, which is in line with the recommendations of the reviewers. In my view, the reviewers are clear and specific, and will hopefully present a resource towards providing a revised version of the paper for further evaluation. Only some minor changes are needed, with inclusion of supporting blot analysis data as supplemental information as requested by reviewer 2. Once you've had a chance to consider the reviewers' comments, please let me know if you have any questions.

We forgot to mention that the Northern blots had been uploaded onto Figshare. We have now also uploaded the Western blots and raw data tables.

Reviewer's Responses to Questions

Reviewer #2: The data are sound, however, a major concern is the lack of Western and Northern blots to illustrate the expression levels of the different cell lines in Figures 2, 3 and 4. These data should at least be included in the supplementary data.

They have been uploaded to Figshare.

Reviewer #3: conclusions are perhaps a little short and worthy of further exploration/explanation
A short discussion of the AU repeats has been added.

Reviewer #1: In places, I wasn't sure why various facts were being provided. I suspect the text could be clarified with a further redraft, with the authors asking themselves if they have explained everything they have written.
Reviewer #2: A few minor points that could also be addressed by the authors:

1. Line 217, “in order to find” instead of “in other find” done

2. Line 251, remove one “that” done

3. The authors used cyan fluorescent protein (eCFP) as reporter, but wrote “GFP protein” and “GFP mRNA” in figures 3C and 3D, respectively. Similarly, GFP instead of CFP is mentioned in S2 Table. This needs to be corrected. In addition, the authors must choose between CFP and eCFP throughout the manuscript.

What actually happened is that we were given this gene many years ago by someone from anther lab who told us it was eGFP. Then when my ex-post-doc Esteban Erben was checking some sequences recently he realised it was actually CFP. There are only point mutations so the antibody used is against GFP. We've corrected everything (we hope).

4. In line 287, it is mentioned that “protein levels were assessed for each population by quantitative Western blotting, with ribosomal protein S9 as a loading control”, however the Western blots are not shown in the manuscript and should be included in the supplementary data (see general comment).
They are available at Figshare, the url is provided.

5. In the legend of Figure 3B, reverse blue and red in the sentence “Each dot is a result for an independent clone, with blue and red representing bloodstream (B) and procyclic (P) forms, respectively” to be consistent with the colors used in the figure.

Oops - thank-you

6. In Figure 3A, all lanes showed an additional band of 1.7-1.8 kb detected by the CPF probe, especially in the right blot. In addition, the position of the markers is different between the two panels (see for example the mRNA detected in lanes 1 that is not have the same size in the left and right blots).

True. The gels definitely ran showewhat differently. I checked the originals and measured the lengths again. Actually the difference is 70nt. The gels appear to have run a little differently. In the Supplementary Table the two measurements are now given and the results from different gels are in different colours.

We don't know what the background band is. It might just be cross hybridisation to the rRNA. This is now mentioned.

7. Line 325, “dataset” instead of “datset”. fixed

8. In the second last paragraph of the result section, as well as in the corresponding figure 4, it is not mentioned in which form of parasite the experiments were conducted, PCF or BSF. We can assume it’s in BSF, but it should be clarified.

Done
9. In the first column of figure 4B, which may correspond to the actin control in Figure 3B, one may expect to see an expression level in PCF around 0.5. But no value for PCF is included in this graph. This important control should be included in the figure.

The important point is that the PGK fragment which, in context, decreases expression by about 90%, had only a marginal effect in the new context.

We realised that the results for actin in Figure 3B and those in Figure 4B didn't include some additional replicates. After inclusion of all in Fig 3B the difference between bloodstream and procyclic forms is less marked, though it's still quite surprising since the same 3'-UTR gives no regulation of CAT. The data for the two blots with replicates, with actin in bloodstream forms normalised to 1, are now included in Figure 4B. The precise values are a bit different (also because the densities were re-measured) but the conclusions don't change.

Reviewer #3: One typo spotted I think, otherwise very clear.

--------------------

Summary and General Comments

Reviewer #1: This study has delineated the 3'UTRs of two transcripts in Trypanosoma brucei that are developmentally regulated and encode important bloodstream-stage proteins. They have succeeded in producing a more accurate map of the regulatory motifs within the 3'UTRs, showing that they are likely bound by many different regulatory proteins. This is a useful achievement that progresses our understanding of the interaction hierarchies that must control development within these parasites. 

I have no direct experience of the methods used in the study, but they are certainly orthodox for the field and well described in the manuscript. I have no reservations about the quality of the work or the veracity of the findings. I have just three comments to make about the text.

First, it begins "Kinetoplastids are unicellular flagellated parasites that infect mammals and plants." I would suggest changing this since most Kinetoplastids are not parasites (better to use 'trypanosomatids') and trypanosomatids largely infect insects and vertebrates (although plants are included). "Mammals and plants" is too selective to be accurate.

True, fixed.

Second, ll194-6 describe an acetyl-coA synthetase coding region downstream of the RBP10 gene. The authors state that "[it is] present elsewhere in the genome, but the presence of read alignments over the region that surrounds the Tb927.8.2790 coding region suggests that the pseudogene mRNA is also present in both bloodstream and procyclic forms." I didn't understand why we were being told this. Is the pseudogene implicated in the RBP10 3'UTR? Please explain what we need to know about this.

We don't, the section has been deleted. (It was relevant in a previous rather different version of the paper.)

Third, in the section beginning on l319 the authors look for common sequence motifs among their regulatory sequences, but find none. Is a similar exercise not possible for secondary structures? Have the authors or others not looked for conserved loops or folds among these UTRs with similar expression profiles?

Unfortunately here still isn't a good way to do this. Moreover, secondary structures of RNAs in vivo are strongly influenced by the proteins that are bound. Doing this will have to await *in vivo* studies of RNA structure on a transcriptome-wide scale. In fact, most mRNA-binding proteins studied so far bind to single strands but of course there are exceptions. However we suspect that similar work to ours on several more 3'-UTRs will be needed in order to reveal motifs. At least, the work we have done narrows it down.

Reviewer #2: The 3'-UTR lengths in trypanosomes is remarkably long compared to other Opisthokont model organisms, with mRNAs having 3'-UTRs of 5 kb or more. This is particularly true for mRNAs encoding RNA-binding proteins, which have vital regulatory functions. As their expression has to be particularly tightly controlled, the authors hypothesized that these long 3'-UTR may contain several regulatory sequences acting independently. To address this question, Tania Bishola Tshitenge and colleagues used two report systems (CAT and eCFP) to identify the regulatory sequences within the 3’-UTR of two mRNA that are responsible for the specific expression in the bloodstream forms of T. brucei (BSF), i.e. the 7.2 kb 3’-UTR of RBP10, an RNA binding protein previously characterized by the same research team, and the terminal 424 nt of the PGKC 3'-UTR that the authors previously identified as responsible for specific expression in BSF. They found that PGKC has at least two such
sequences, while RBP10 has at least four which are scattered throughout the 7.2 kb 3’-UTR. The authors concluded that several different sequence motifs - and therefore, probably, a similar number of RNA-binding proteins - are implicated in controlling bloodstream-form-specific mRNA stability and translation.
The data are sound and the conclusions drawn by the authors are consistent with the data presented. However, a major concern is the lack of Western and Northern blots to illustrate the expression levels of the different cell lines in Figures 2, 3 and 4. These data should at least be included in the supplementary data.
These are now all at Figshare.

Reviewer #3: I went through this line by line, comments and questions given below with their line numbers.
48/50/51/52 PGK, systematic gene names the first time the gene is mentioned please.
Done

70: quantitate "much more" This was left vague because the results are pretty divergent, but we have now given a number.

75: RBP6 systematic name? done

88: Opisthokont, is this not a rather diverse group about which to make comparative comments? No, what we actually say is "Opisthokont model organisms", of which there are really only five: yeast, humans, mice, C. elegans and D. melanogaster. And the comment is true for all of them.

94: systematic name for ZC3H22? done

96-97: what is meant by "better expressed"?  Not quite clear here, no numbers either.

The original sentence was : "Over 400 *T. brucei* mRNAs are at least 10-fold better expressed in bloodstream forms than in procyclic forms (as judged by ribosome footprints)."

This has now been expanded to " as judged by the relative numbers of ribosome footprints, which measure of both mRNA abundance and translation"

98: give the 8mer sequence Done

103-105: maybe actually give the sequences here for the reader!?

The sequences were already in the supplement and this was mentioned in the text.

129: are the RBP10 3' UTR sequences identical in 427 and 1125?  Was there a reason not to use

1125 genomic DNA as a template for the PCRs?

At the time the EATRO1125 sequence was not available, but now it is and the identity is 97% - and some of the differences are in repeated regions.

The important point for this work is that the sequence that we used works.

135-136: very ambiguous about how much of what was actually sequenced to confirm identity: this is important.

As the section says, the sequences of all clones and the alignments are included as supplementary Figure S1.

188-192: I found this a bit confusing: are there TWO regions of UTR like sequence in separate locations in the genome, and if so, how can you be certain which one is responsible for the noted effects?  Are there not PacBio sequence data available for 1125, which might help clear up this quandry, given it is perhaps central to how robust your conclusions/inferences might be?

Actually we are confused too. We have no idea why this sequence is grey in the alignment. We can't find the other copy. The sentence has been changed to:

"The middle region of the 3’-UTR is shown as being present more than once in the TREU927 genome assembly, as indicated by the grey-coloured reads in the alignment in Figure 1A, but we have been unable to locate this other copy in either this or the other available genomes.

206-207: see 188-192?

Yes, text changed to:

"When we extrapolated the marker curve, the results gave an *RBP10* mRNA length consistent with the 8.5 kb from RNASeq."

Fig 1A, units for Y axis?

The absolute numbers aren't important, since coverage is different for every dataset. Instead the salient point is to compare the RBP10 reads with those of neighbouring genes.

Fig 1A, procyclics, wild/crazy observation, but it looks as if majority of transcripts may not have the long UTR sequence for 2780, perhaps polyadenylated much closer to the stop codon?  Do you have any oligo-dT based 3' end sequence/PCR data for checking this out?  Or ratio of read depths for the CDS and 5UTR in B or P on basis that if it has the CDS it should have the UTR.  Might fit with your comment on line 232.  Might depend on the characteristics of the RNAseq libraries whether that is meaningful; the sequence reads used in Seattle were pretty short and were aligned locally using bowtie2; are there no more recent data with larger insert, paired end reads?

If you look at other datasets there is no indication of earlier polyadenylation. For example, the Qi et al results from cells grown without added glucose (like ours) show coverage right across. Oligo d(T) based results are unfortunately uninterpretable for this UTR because it has multiple poly(A) tracts. Even nanopore won't manage the whole mRNA very often. In fact, the gold standard for this is still the Northern blot - see Figure 1B!

Fig 1D, lower panel: two? sets of blue dots, are they the three replicates?, no error bars

The legend now says:

" The graph shows quantitation (individual results) from three independent experiments, the horizontal line is the arithmetic mean."

209-210: range, SD of measurements? Saying "probably" without stats is meaningless!
It is impossible to obtain valid standard deviations from three replicates since the basic requirement (a normal distribution) cannot be demonstrated. Since error bars would be invalid, as would t-test values, they are not shown. Non-parametric tests don't work on less than 5 replicates.

We know that using these invalid measurements has been standard for decades but prefer not to do it any more.

217: typo
Fig 2: no indication what the different colours indicate? I think they are regions/constructs, but we dont see them all together until Fig 5

We have added " Different segments are in different colours and the diagram is to scale." Also the full-length cartoon has been added.

Fig 2: should show the low-complexity repeat regions

We can't do this because we have no idea what threshold to apply - and there is, so far as we know, no standard. Readers can judge for themselves, the sequences are in the supplement.

Fig 2: For "expression", averages are: "-" = <0.25x, "+/-" = 0.25-0.5x, "+" = 0.5-2x, "++"=2-3x and "+++" >3x.  I have "problems" with these categories, with values less than 1 being included in the "+" category, surely that would be either "-" or if redefined, "+/-" perhaps?

The boundaries of the categories are of course arbitrary. What is important is to be transparent - and the detailed data are in the Figure,

241-242: why did you not just use the means, would be much more appropriate?  Currently a very curious choice of metric that artificially maximises the result divergence, particularly pronounced when n is such a small number...

Medians exclude big outliers, whereas arithmetic means include them.

243: "mostly not normal": by what measure did you reach this conclusion?

Indeed, we cannot. Changed to

"because with 3 replicates, it is impossible to tell whether the values are normally distributed;"

248: use nuclease digestion to make fragments?

This would be possible, but we chose the PCR approach.

261: what are the sequences of the regulatory elements proposed?

All of the fragment sequences were already in the supplement, as stated in the text:

" The sizes of the mRNAs are tabulated in Supplementary Table S2 and the sequences are in Supplementary text 1."

274: was this approach done for RBP10 at all?

No, most of the pieces are still too long for reliable predictions.

278: was the RNAfold re-run on these deleted lines, to check that things were still as you expected/predicted?

Yes, but predictions require experimental verification, which was not done.

293-294: this is possibly worthy of further explanation/exploration?

Probably not - at least in the context of this manuscript. At least we checked, many people just do qPCR!

300-301: is the variability seen across any other mRNAs you measured (if you did)

The RBP10 results (see Figure) are actually just as variable. Ideally one should make five individual clones for each deletion but that was beyond our capacity.

303: which is...?

We don't know. This is inter-clonal variation which could be caused by multiple different factors, including quite minor differences in cell density. This phrase has been deleted.

331-340: were RNAfolds done for all the various fragments to try and draw a link to the observed experimental results?

No, since predictions would require *in vivo* verification anyway.

331-340: these are all longer than what the MEME search was done with, so you may be missing things

If so, they would be bound by multiple proteins. In fact, it doesn't matter how you do the search, nothing is found. We've done it multiple times over the last few years, and had hoped that the results from PGKC would help. Sad that they didn't, but with more analyses of other UTRs probably something will emerge.

331-340: what other genes have similar AU repeats in their 3'UTR (or, for that matter, perhaps also the 5UTR?) We looked at this and found 580; mRNAs which have them have longer 3'-UTRs than those without, so the specificity is a bit questionable. The number has now been added to the results section.

362-365: perhaps, but what are the sequences in the fragments?  Are there any footprint data for these regions from other studies?

As stated in the paper, all of the sequences were already in the supplement. Unfortunately, there are no publicly available RNA footprint results for trypanosomes. Summaries of CLIP-Seq results for RBP42 and DRBD3 were published but the locations of the footprints were never provided and the raw data are not available - we've asked! The publication preceded current data transparency requirements.
